# Impact of PP Impurities on ABS Tensile Properties: Computational Mechanical Modelling Aspects

**DOI:** 10.3390/polym13101647

**Published:** 2021-05-19

**Authors:** Charles Signoret, Anne-Sophie Caro-Bretelle, José-Marie Lopez-Cuesta, Patrick Ienny, Didier Perrin

**Affiliations:** 1 Polymers Composites and Hybrids (PCH), IMT Mines Ales, 30100 Ales, France; charles.van.signoret@orange.fr (C.S.); José-Marie.Lopez-Cuesta@mines-ales.fr (J.-M.L.-C.); Didier.Perrin@mines-ales.fr (D.P.); 2 LMGC, IMT Mines Ales, Université Montpellier, CNRS, 30100 Ales, France; Patrick.Ienny@mines-ales.fr

**Keywords:** polymer recycling, WEEE, predictive modeling, volume change

## Abstract

Recycling of plastics is hindered by their important variety and strong incompatibility. However, sorting technologies bear costs and meet limits. Very high purities (<2 wt%) are difficult to reach. Yet, such rates may be detrimental to functional properties. In this work, an ABS matrix (major plastic in Waste of Electrical and Electronic Equipments) was filled with 4 wt% of PP to mimic impurities in ABS after recycling. PP-g-MA was introduced in the blend to improve the compatibility. A finite element model was developed from the mechanical behavior of each component. ABS and PP were individually characterized from tensile tests instrumented with photomechanics and their behaviors were modelled through a set of numerical parameters (elasto-visco-plasticity with a Gurson’s criterion behavior). Comparison between the determinist model results and the experimental data (strength, volumetric variation) shows that this type of modelling could be a predictive tool in order to anticipate composite mechanical properties and to understand micromechanisms of deformation (damage, cavitation). The main result is that PP introduced at 4 wt% into ABS does not alter the static mechanical properties despite polymers incompatibility. The addition of PP-g-MA modifies the local properties and possibly conduct to a premature breakage of the polymer blend.

## 1. Introduction

Waste of Electrical and Electronic Equipment (WEEE) Plastics (WEEP) are not currently intensively regenerated through mechanical recycling for several different reasons [1,2]. Dismantling these devices is a complex subject [3,4]. Moreover, the rapid increase of tonnages [5], the potential toxicity of these products [6], and the presence of valuable materials as precious metals [7] strongly motivate the establishment of proper end-of-life management scenarios. Strong occurrence of toxic and/or environmentally harmful and now forbidden (or soon to be) brominated flame retardants within polymeric parts [8] is also a serious barrier to waste management as it is unconceivable to reemploy these contaminated materials [9]. High purity rates of sorted polymers are probably unattainable, especially as purity and yield are often contradictory notions [10]. However, even a few percent of impurities can be detrimental to mechanical properties [11] as most polymers are incompatible. In the area of 3D printing, recycling plastics has become increasingly important as this technology can produce large quantities of polymer wastes (support structures, parts with defects) [12,13,14].

As one of the most present polymers within WEEE [15,16] and owing to its potential added value because of its properties, ABS (acrylonitrile-butadiene-styrene) is here considered as the material to be valorized. As the most present plastic after styrenics (ABS, HIPS and their blends, ABS/PC, HIPS/PPE…) and bringer of strong incompatibility because of its different nature, PP (polypropylene) was chosen for the role of contaminant. Some studies were reported about ABS/PP blends, focusing on PP-rich systems or exploring the whole range by 10 or 20 wt% steps [17,18,19,20,21,22,23,24,25]. Apart from strain at break, most of ABS properties are seriously impaired with PP incorporation. The major point is the weak interaction between ABS and PP due to their chemical differences leading to poor mechanical properties. Several compatibilizers were tried but they improved properties mainly in PP-rich zones, performances still far below pure ABS [19,20,21,24].

The aim of this study is to improve the comprehension of the micromechanics of deformation involved during a tensile test with PP added to ABS at a low weight fraction with or without PP-g-MA. A predictive model associated with tensile test and optical instrumentation is introduced. From the identification of the mechanical behavior of each phase, the Gurson-Tvergaard-Needleman (GTN) model [26,27,28] has been used to describe the elasto-visco-plastic and damageable behavior of ABS and PP matrices. Knowing the microstructure, a predictive modeling based on finite element analysis (FEA) was conducted to understand the local phenomena leading to blends breakage.

The paper is structured as follows: Section 2 presents materials and experiments; Section 3 highlights modelling; Section 4 discusses about results; and Section 5 brings a conclusion.

## 2. Materials and Methods

### 2.1. Materials

Virgin unformulated polymer references based on Terluran GP22 (Styrolution) ABS and PPH 9020 (PP Homopolymer-Total) were used for this study. With the aim to improving interfacial interactions, Bondyram 1001 PP-g-MA provided by Polyram Plastic Industries (Ram-on, Israël) was used.

**Figure 1:**
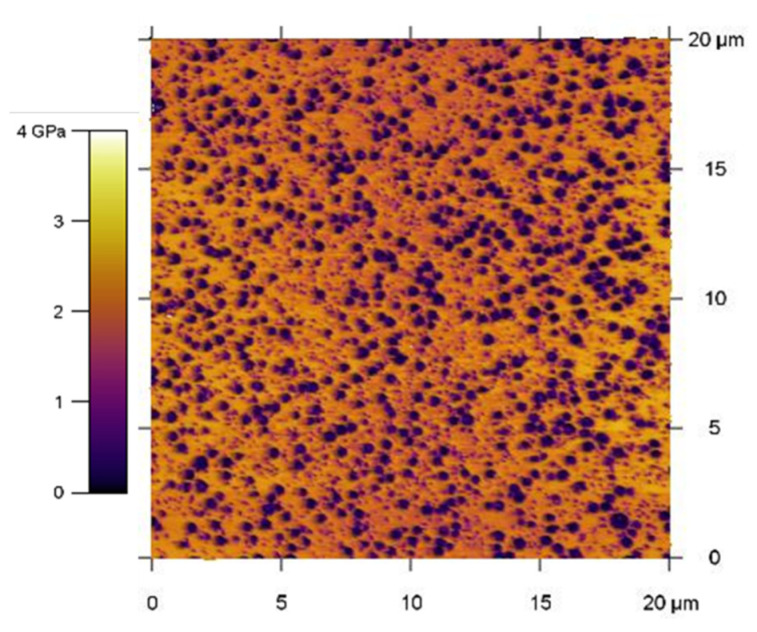
Nodular dispersion of PB within ABS—AFM picture produced by combination of topography and modulus cartography owing to AM-FM mode.

ABS is biphasic by nature. SAN, a statistical copolymer of styrene and acrylonitrile often at 25 wt% [29]), is its majority phase. However, this amorphous polymer is very brittle and thus unsuitable for many applications. Thus, polybutadiene (PB) rubber is present as a nodular minority phase, as shown by AFM cartography on Figure 1, to improve its impact properties. For common applications, PB loading rates are reported between 10 and 30% within ABS depending on if flow properties or impact resistance is desired [29]. For emulsion grafting, the main synthesis route for commercial ABS, size distribution is reported between 0.1 and 1.0 µm, but mainly with a median diameter around 0.3 µm [29] (Figure 1). PP is also biphasic in another way as it is semi-crystalline, meaning that crystallites, where chains are spatially organized, are dispersed within an amorphous phase.

ABS and PP were mixed at 4 wt% since previous works from our laboratory [30] highlighted that properties loss began at this rate. PP-g-MA was tried at 3 phr (parts per hundred of resin) to improve interfacial adhesion between phases.

ABS pellets were dried at 80 °C for at least 16 h before processing. ABS and PP were manually mixed at desired ratio before extrusion within a 900 mm Clextral twin-screw extruder of type BC21 at 250 rpm, 220 °C along the screw, with a 5 mm nozzle, at a 6 kg/h feed speed owing to a K-Tron KQx-2 weighing feeder from Coperion. Extrudate was then pelletized owing to an SGS-E50 from CF Scheer & Cie (Stuttgart, Allemagne). For mechanical properties, Type A dogbones specimen corresponding to ISO 3167, was injected using a Krauss-Maffei 180/CX 50 molding injection press at 230 °C.

### 2.2. Methods

Uniaxial tensile tests were performed on an MTS criterion model 45 universal testing machine (Eden Prairie, MN, USA) following ISO 527 standard on above-mentioned specimen. Tests were conducted at 1 and 10 mm/min (corresponding respectively to strain rates 10^−4^/s, 10^−3^/s) to highlight possible time dependences. Specimens were brought to 1% strain, unloaded, and then brought to break. The software used is TestXpert^®^ (Ars-Laquenexy, France) and allows the recording of time, load, and elongation. During these works, x is defined as the tensile direction, y perpendicular to x and in the planar surface, and z the out of plane direction.

The axial nominal stress is given by the following expression (Equation (1)):

(1) $$\sigma_{x}=\frac{F}{S_{0}},$$

where *F* is the recorded load and *S*_0_ the initial sample section surface.

**Figure 2:**
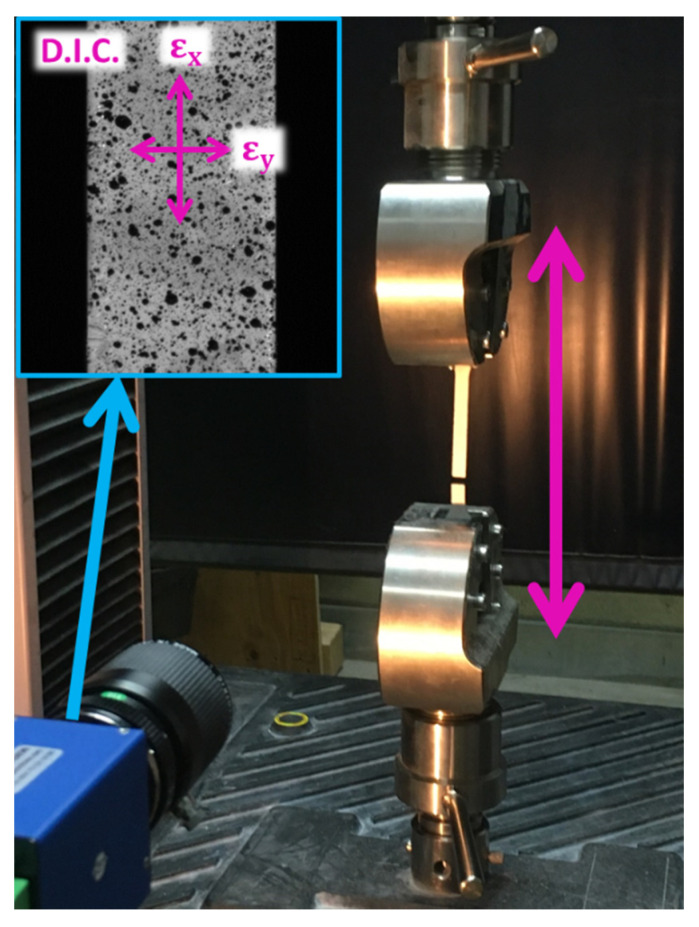
Testing apparatus.

The optical extensometer involves a high-resolution charge-coupled device (CCD) camera (Redlake Megaplus II (Tuscon, AZ, USA), 1920 × 1080 contiguous and square pixels, coded in 256 grey levels), set in front of the specimen, which records images during the test. The optical axis of the camera remains perpendicular to the in-plane surface of the specimen during the test (Figure 2). The images acquisition is commanded by the LabVIEW^®^ software (National Instruments, Austin, TX, USA) which allows the simultaneous acquisition of the images and the data from the testing machine (such as load and crosshead displacement). According to the test speed used, images are recorded every 0.5 s. The scale factor is fixed to 42 µm per pixel.

The in-plane Green Lagrange strains $E_{x},\;E_{y}\;$were deduced from digital image correlation (DIC) following a method well described in previous works [31]. A transverse isotropic assumption was considered and validated as in a previous study on SEBS specimen submitted to uniaxial tensile test [32].

The volumetric strain is defined using front-view data only and assuming isotropy:

(2) $$\frac{∆V}{V_{0}}=\lambda_{x}^{2}\lambda_{y}-1\;\mathrm{where}\;\lambda_{i}=\sqrt{2E_{i}+1},\;i=x,y,$$

*V* is the current volume, *V*_0_ the original volume, and $\lambda_{x}\;$and $\lambda_{y}$ are the in-plane principal stretch ratios in the *x* and *y* direction. The part of elastic volumetric strain can be computed (Equation (3)):

(3) $${\left(\frac{∆V}{V_{0}}\right)}_{elas}=\frac{\sigma_{x}}{E}\left(1-2\nu \right),$$

where *E* and *ν* are the elastic material parameters (Young modulus and Poisson’s ratio).

## 3. Results

### 3.1. Mechanical Characterization

#### 3.1.1. Mechanical Characterization of ABS and PP

ABS and PP behave mechanically quite differently. The first polymer is brittle in comparison to the second one. During the first loading/unloading cycle until 1% of strain, the elastic properties (*E* and *ν*) of virgin polymers are computed for both imposed speeds (see average values with standard deviation in Table 1). Regarding elastic properties, PP seems more impacted by the speed rate, especially with respect to the Poisson’s ratio, with an increase of 10% while increasing the speed from 1 to 10 mm/min.

**Figure 3:**
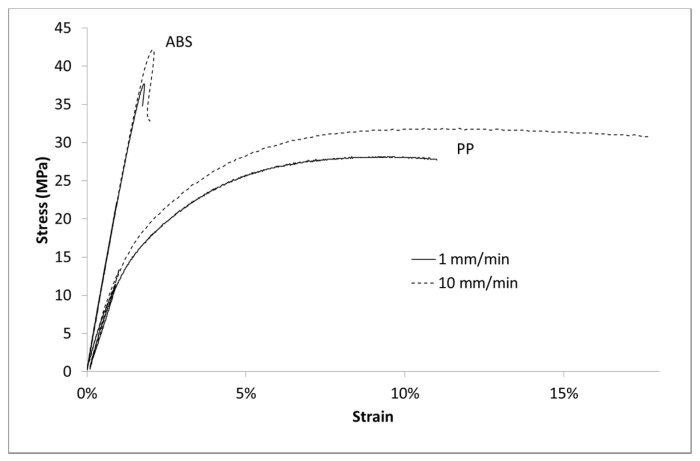
Stress vs. Lagrangian strain in uniaxial tensile tests at several imposed strain rates for ABS and PP.

**Figure 4:**
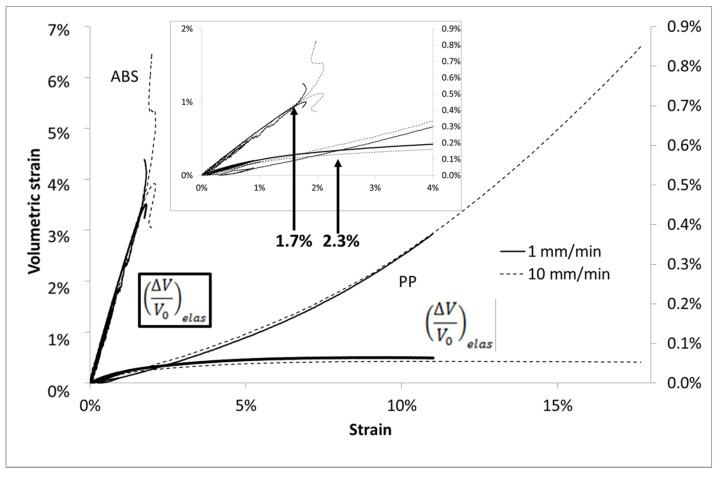
Volumetric strain vs. Lagrangian strain in uniaxial tensile tests at several imposed strain rates for ABS and PP, focus on strain until % to enhance the deviation point at 2.4% from elastic behavior for PP sample.

Moreover, the ABS matrix behaves as a damageable elastic material. Its global behavior is time independent (Figure 3) and its volumetric strain (deduced from Equation (2)) follows an elastic law (deduced from Table 1 and Equation (3)) until 1.7% of imposed strain (Figure 4). However, PP polymer behavior is more sensitive to the strain speed with regard to the stress values. Moreover, the volumetric strain deviates from the elastic curve from 2.3% of strain and is time independent. For a same level of strain, the stress curve is very different for both polymers whereas the volumetric strain is almost similar.

#### 3.1.2. Mechanical Characterization of Composites

**Figure 5:**
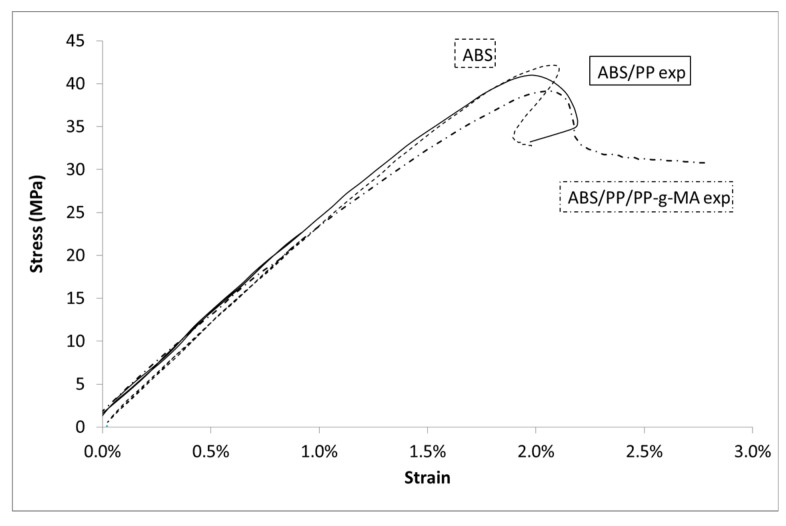
Stress vs. Lagrangian strain in uniaxial tensile tests at 10 mm/min for ABS, PP, and composites.

**Figure 6:**
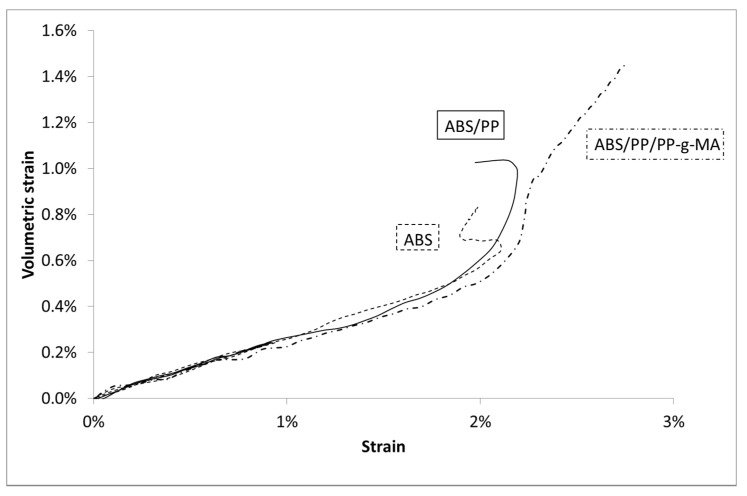
Volumetric Strain vs. Lagrangian strain in uniaxial tensile tests at 10 mm/min for ABS, PP, and composites.

Once PP is added into ABS matrix at 4 wt%, the global shape of the stress/strain curve is similar to virgin ABS (see for example Figure 5 for the higher speed test). As expected, the Young’s modulus is lowered by the introduction of a less stiff polymer just like the associated maximum stress. This phenomenon is enhanced by the introduction of PP-g-MA (see Table 2). The volumetric strain (Figure 6) is almost unchanged as PP is introduced into ABS with or without the presence of PP-gMA. The introduction of PP impurities into ABS does not impact ABS volumetric strain, even if the stress/strain curve is slightly lowered as PP-g-MA is introduced into ABS/PP.

### 3.2. Morphology Characterization

Morphologies were monitored owing to SEM (scanning electron microscopy) (Quanta FEG 200, Thermo-Fisher Scientific, Waltham, MA, USA) on cryofractured dogbones (orthogonally or 45°) and post-mortem Charpy impact fragments. To produce cryofractured dogbones polymer samples were first immersed into liquid nitrogen for approximately 5 min and then taken out and struck with a hammer. AFM (Oxford Asylum Research MFP3D, Santa Barbara, CA, USA) was also performed on samples prepared using a Leica EM UC7 ultracryomicrotome (Leica, Nanterre, France). AM-FM (amplitude modulation-frequency modulation) mode of AFM was applied to enable mapping of local pseudo-moduli, enabling easier differentiation between SAN and PB as their respective moduli are different.

#### 3.2.1. Nodular Dispersion of PP within ABS

**Figure 7:**
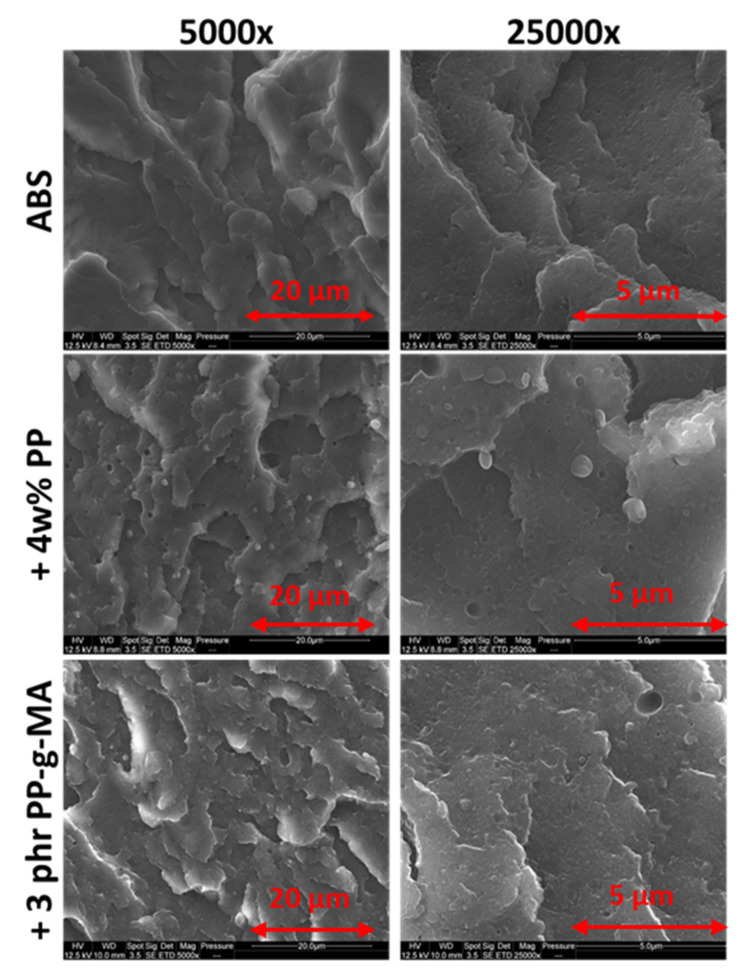
Nodular morphology of PP within ABS at 4 wt% with and without PP-g-MA- two magnifications.

**Figure 8:**
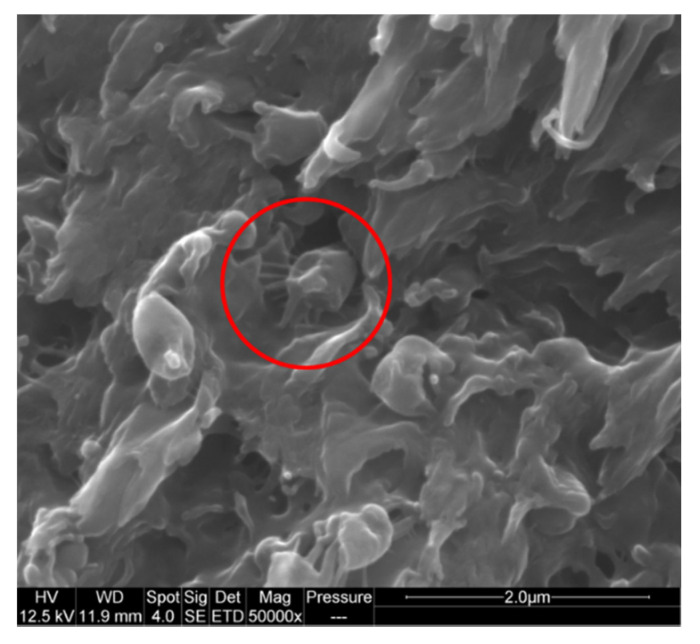
SEM picture of cross-sections of ABS-PP-PP-g-MA samples after tensile test—fibrils linking a potential PP nodule to the matrix.

As expected, polypropylene forms a nodular dispersion within ABS as Figure 7 shows. Cryofractures prove that PP nodules adopt a spherical shape and not an ellipsoidal or cylindrical one. Moreover, PP nodule sizes are reduced with PP-g-MA addition. A possible adhesive interaction is visible in Figure 8 as fibrils are seen between what could be a PP nodule and the matrix. However, this micrograph corresponds to a very specific area of the fracture and was not found elsewhere. Part 2.4.2 below presents micrographs more representative of the post-mortem samples.

**Figure 9:**
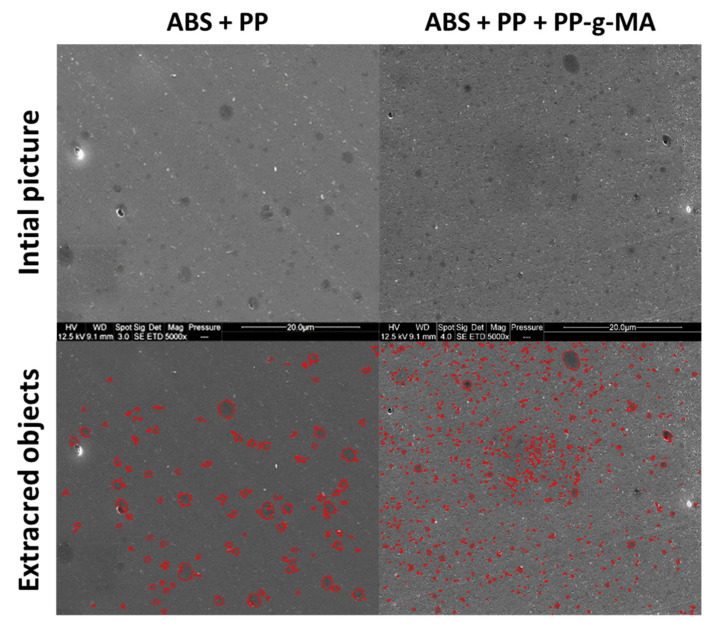
Binarization of SEM micrographs of cuts performed using an ultracyromicrotome—ABS + PP and ABS + PP + PP-g-MA systems.

**Figure 10:**
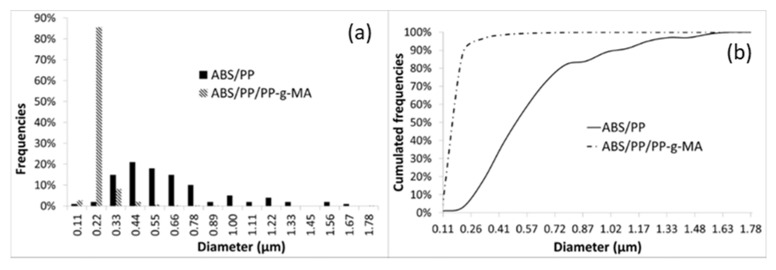
Frequencies (**a**) and cumulated frequencies (**b**) of diameters sizes distribution for ABS/PP and ABS/PP/PP-g-MA composites.

Size distribution of nodules was determined by image analysis (AphelionTM 3.2 (ADCIS)). SEM micrographs were binarized and PP nodules were identified and filtered by surface area (see Figure 9). SEM pictures on samples cut with an ultracryomicrotome were preferred as micrographs issued from cryofractures were too uneven to enable image analysis. AFM pictures were also more complex to analyze as both PP and PB were visible. Each identified object is assumed to have a disk shape well described by its diameter. ABS/PP and ABS/PP/PP-g-MA were analyzed owing to this procedure. Around 800 PP nodules were identified for both compositions with nodules diameters from 0.2 to 1.7 µm for ABS/PP blend and from 0.1 to 1.0 µm for ABS/PP/PP-g-MA blends (Figure 10).

#### 3.2.2. Cavitation Phenomenon

**Figure 11:**
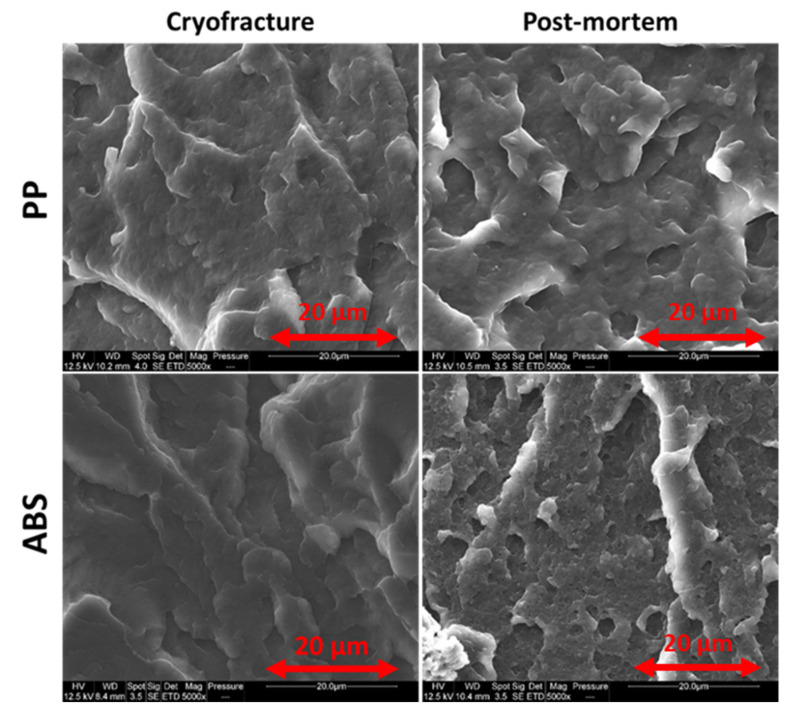
Comparison of cavitation phenomena within ABS and PP—SEM pictures on cryofractured dogbones and post-mortem samples from tensile tests at 10 mm/min.

Figure 11 compares the formation of porosity or cavities on the broken cross section within ABS and PP after tensile tests pursued until failure. These pictures show porosities/cavities areas which seem larger in the case of PP but this polymer came to break at higher strains in comparison with ABS.

**Figure 12:**
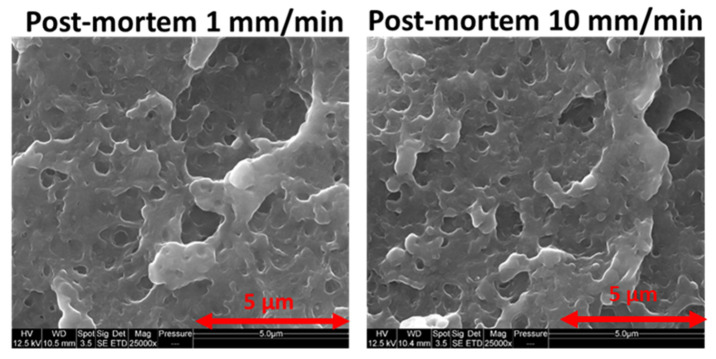
ABS cavitation independence toward strain speed—SEM pictures of post-mortem samples.

Figure 12 shows that the cavitation process related to PB particles is very similar within virgin ABS even with different strain speeds. This is in accordance with the observations of Figure 4 for which volumetric strains of virgin polymers are insensitive to the imposed speeds.

**Figure 13:**
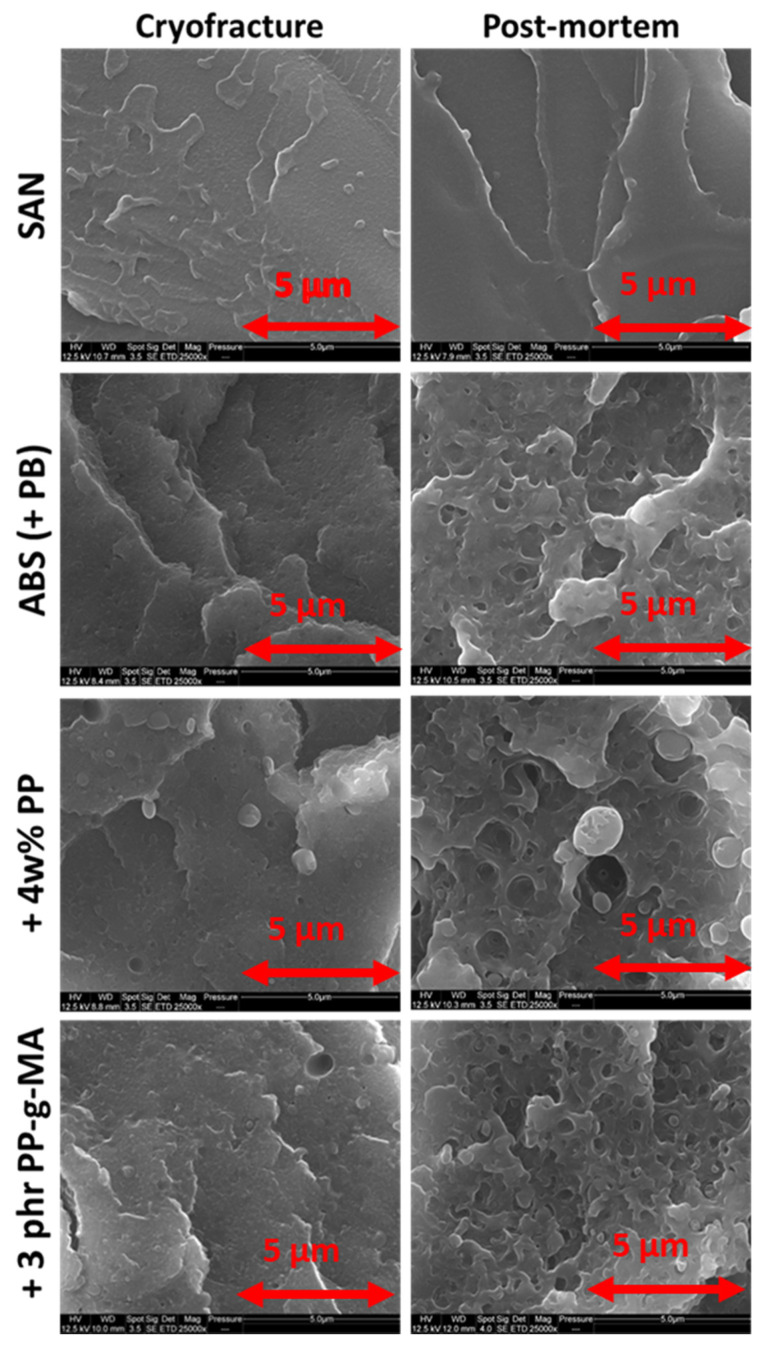
Cavitation phenomena observed on post-mortem tensile tests fragments through SEM—comparison to cryofractured samples—virgin SAN, virgin ABS, and ABS contaminated with 4 wt% PP and ABS contaminated with 4 wt% PP with 3 phr of PP-g-MA.

Figure 13 shows cryofractures and post-mortem samples of SAN, ABS, ABS/PP, and ABS/PP/PP-g-MA. As a fragile material, SAN does not form cavities and fractures are thus very similar from cryofracture to post-mortem. As reported in the literature [33], PB generates cavitation within ABS, enabling an improvement toward fracture as it dissipates energy. Smallest holes on the picture are about 0.2 µm in diameters and biggest ones at 1.4 whereas most of them are around 0.4–0.5 µm. PP presence worsens dramatically the phenomenon as most holes seen here are more around 1.0–1.5 µm. With PP-g-MA the hole size is around 0.5–1 µm, closer to pure ABS. This is surely due to the morphological refinement described in Figure 10.

### 3.3. Modelling

#### 3.3.1. Behavior

The literature reports an important number of damage models, able to account for mechanical degradation in materials. Possibly, these models include new internal variables and evolution laws coupled to plasticity problems [34]. The macroscopic measured volume change is assumed to be related to cavitation occurring within the polymers phases by void nucleation and growth [35]. The present work focuses on a Gurson-type approach which is based on mechanics of porous media. The damage indicator, related to the degradation of the material, is the void volume fraction (i.e., the porosity) which progressively downscales the yield surface. In the early eighties, Tvergaard and Needleman [28,36] extended the Gurson approach, namely GTN model, including material hardening, multiple voids, and void coalescence. This GTN model, available in numerous finite elements software, has been already used by authors to describe the micromechanisms of deformation of a PP/Bakelite blend [37] for which the PP matrix exhibits porosity following a Gurson-type model.

In this damage formulation, the yield criterion uses hydrostatic pressure and porosity: the criterion of plasticity and the plastic potential, depend on a macroscopic stress $\sigma_{*}\;$and an effective porosity fraction *f_t_* in the following way (Equation (4)):

(4) $$\frac{3J_{2}\left(\tilde{\sigma }\right)}{\sigma_{*}^{2}}+2f_{t}q_{1}cosh\left(\frac{q_{2}I_{1}\left(\tilde{\sigma }\right)}{2\sigma_{*}}\right)-\left(1+q_{1}^{2}{f_{t}}^{2}\right)=0,$$

where $\tilde{\sigma }$ is the stress tensor $J_{2}=\frac{1}{2}dev\left(\tilde{\sigma }\right):dev\left(\tilde{\sigma }\right)$, $I_{1}=trace\left(\tilde{\sigma }\right)$ and $q_{1},$ $q_{2}$ are model parameters. The $q_{1}$ parameter allows to adjust the influence of *f_t_* on the yield surface and the $q_{2}$ parameter is related to the pressure effect.

Effective stress $\sigma_{*}$ is implicitly evaluated from Equation (4).

The plastic flow potential is written as (Equation (5)):

(5) $$f=\sigma_{*}-R\left(p\right),\;\;R=R_{0}+Q_{1}\left(1-e^{-b_{1}p}\right),$$

where *p* is the effective plastic strain and $R_{0},Q_{1},\;b_{1\;}$ are material parameters.

The evolution of viscoplastic strain is written through a Norton’s law:

(6) $$\dot{\tilde{\varepsilon }}=\left(1-p\right)\dot{p}{\tilde{n}}_{*},\;\;{\tilde{n}}_{*}=\frac{\partial \sigma_{*}}{\partial \tilde{\sigma }},\;\;\dot{p}={\left(\frac{f}{K}\right)}^{n},$$

where *n*, *K* are material parameters.

The total porosity change rate can be split into two components, one attributed to the void growth ${\dot{f}}_{g}$ and the other one to the nucleation ${\dot{f}}_{n}$:

(7) $${\dot{f}}_{t}={\dot{f}}_{g}+{\dot{f}}_{n},$$

where ${\dot{f}}_{g}=\left(1-f_{t}\right)tr\dot{\tilde{\varepsilon }}$ and ${\dot{f}}_{n}=A\dot{p}$, *A* is a constant.

##### 3.3.2. Determination of ABS and PP Model Properties

**Figure 14:**
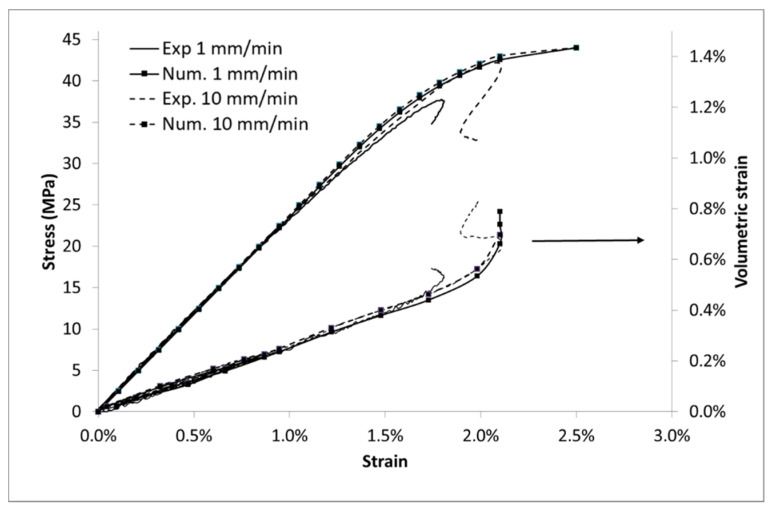
Modelling of ABS, stress and volumetric strain vs. Lagrangian strain.

**Figure 15:**
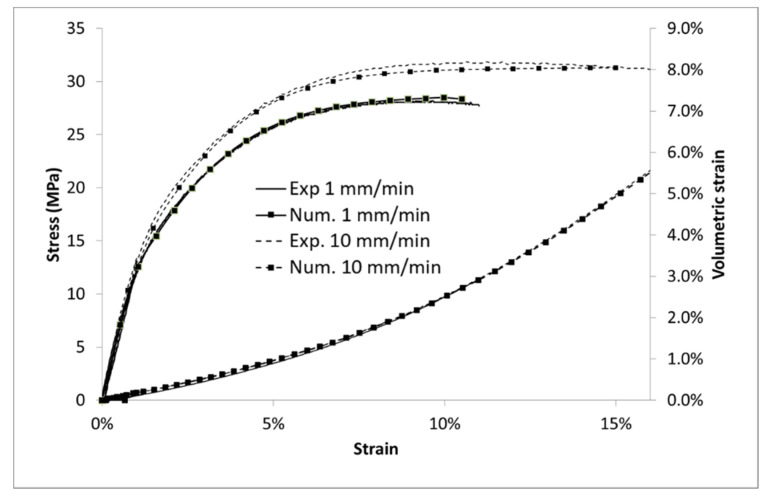
Modelling of PP, stress, and volumetric strain vs. Lagrangian strain.

The behavior of PP and ABS can be well described through this modelling. The Young’s modulus *E*, the Poisson’s ratio *ν* and the yield stress *R*_0_ were determined from stress/strain data. The GTN model has been implemented using the Matlab software. The porosity evolution parameters $q_{1}$ and $q_{2}$ are arbitrarily fixed to 1, adjustments of these values would necessitate some local data (as porosity distribution) [35]. The GTN model is therefore reduced to the native Gurson model. The remaining material parameters were identified by using a mean square method on stress/strain and volumetric strain/strain data between experiments and numeric data at each timestep of the simulation. Parameters present in Table 3 lead to a good accordance between experiments and modelling for both stress (Figure 14) and volumetric strain (Figure 15). The contrast on the K value prevails for the low time plasticity dependency for the ABS.

#### 3.3.3. Prediction of ABS/PP Blend Properties

For the sake of simplicity, a plane stress assumption is made for the numerical computations even if this strong hypothesis is far from reality. The interface between matrix and fillers is assumed to be perfect (displacement continuity) and phase properties are described through the set of parameters of Table 3. This assumption of perfect interface will be discussed in next section as the compatibility of ABS and PP is quite questionable. We assume that the properties of ABS and PP in the blend are similar to those of the neat ABS and PP.

**Figure 16:**
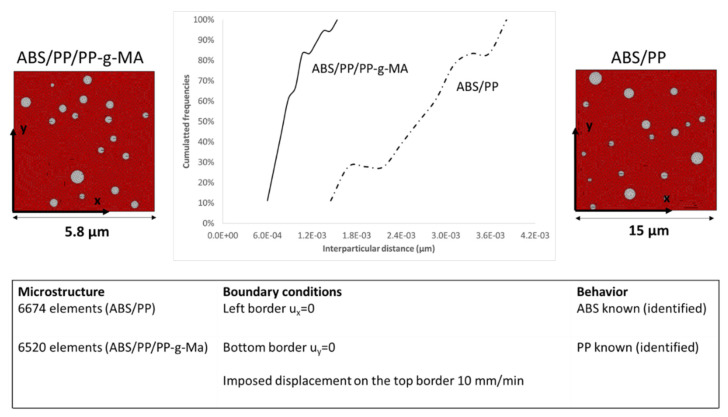
Finite element modelling of ABS and PP blend: structure and boundary conditions.

From the particle size distribution shown in Figure 10, two kinds of microstructures were generated via DIGIMAT software (for both ABS/PP and ABS/PP/PP-g-MA blends). Mesh microstructures based on triangular elements and boundary conditions are presented in Figure 16. The RVE (representative volume element) sizes were chosen to stabilize the macroscopic stress/strain responses. As local behavior is of interest for this study, the chosen mesh refinement leads to stabilize local blend response. We assume the structure symmetry. A prescribed displacement (corresponding to 10% of strain) was applied on the top of the sample. The non-linear numerical simulation was carried out using the FE software Zebulon, which was developed at Mines Paristech [38]. This RVE model is thus useful to predict the mechanical properties of ABS/PP composites. The size of the PP nodules is much lower when a compatibilizer is added: this induces both a reduction in the RVE size and interparticular nodules distances for the ABS/PP/PP-g-MA blend (see Figure 16). We can deduce from these results that interface areas between ABS and PP are increased since nodules are smaller.

## 4. Discussion

**Figure 17:**
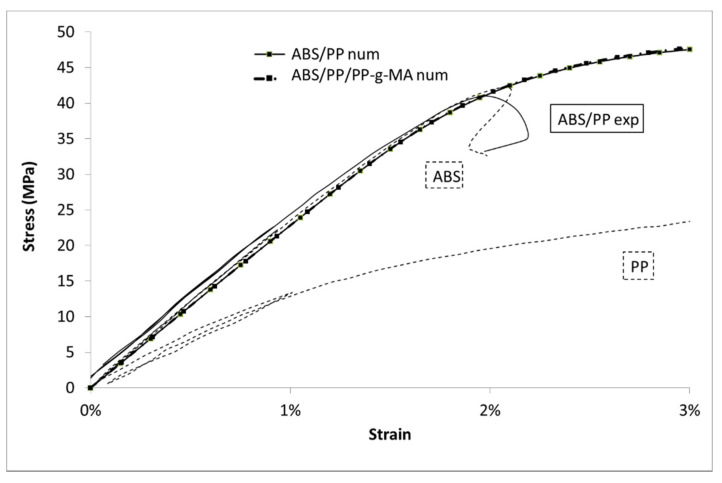
Stress vs. Lagrangian strain for ABS/PP and ABS/PP/PP-g-MA composites, experiments and modelling.

Comparison between numerical models and experimental data for ABS/PP blends is given in Figure 17. The simulated results for both systems (ABS/PP and ABS/PP/PP-g-MA) coincide. As a first result, input materials parameters lead to good predictive modelling as “ABS/PP num” and “ABS/PP exp” are similar until 1.7% of strain. Though unicity of the solution to the model is not proven, the good accordance between experimental results and those predicted by the model assures the validity of the set of parameters used here. After 1.7% of strain, in the experiments, the blends broke quickly. Obviously, this is not the case in the numerical simulations because no rupture criterion has been added.

**Figure 18:**
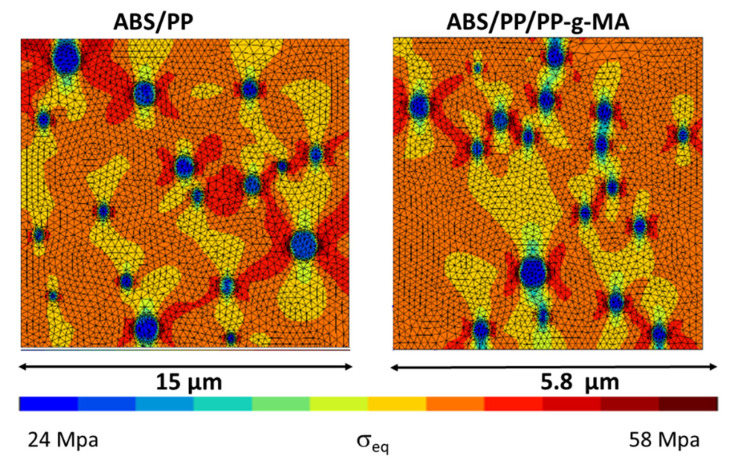
Von Mises stress cartography at 1.7% of macroscopic strain in ABS/PP and ABS/PP/PP-g-MA blends.

A first approach to simulate polymer blends breakdown is devoted to evaluate the equivalent stress (Von Mises) in each element of the RVE. A stress mapping at 1.7% of macroscopic strain is presented in Figure 18 for both blends.

**Figure 19:**
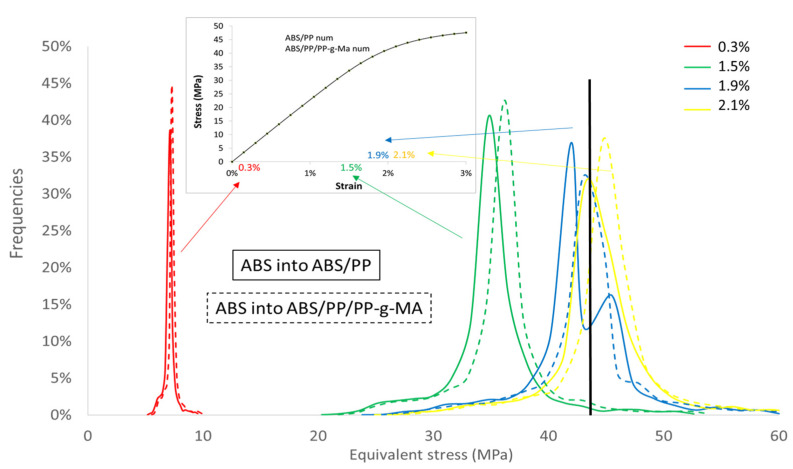
Frequencies of elements versus equivalent stresses for several macroscopic strains for ABS into ABS/PP and ABS/PP/PP-g-MA blends.

To quantify this mapping, we analyzed the Von Mises stress distribution within the ABS. As local behavior is not element size dependent, the comparison between compatibilized and uncompatibilized ABS/PP is possible even if RVE and elements sizes are not equivalent. The Figure 19 reflects the distribution of Von Mises stresses into ABS polymer for several macroscopic strains (0.3, 1.5, 1.9, and 2.1%) for ABS/PP and ABS/PP/PP-g-MA composites. The ultimate stress of ABS polymer is around 43 MPa, beyond which the sample breaks. The main conclusion is that reducing the size of PP nodules by compatibilization leads to a local increase of the level of equivalent stress by the diminution of the interparticular distance between nodules (see Figure 16). The frequency curves are thus shifted to the right.

The number of elements with equivalent stresses higher than 43 MPa (corresponding to the value of ABS breakage) was computed from these data (see Table 4: Percentage of elements in ABS). At 1.9% of macroscopic strain both blends show a large number of broken elements in ABS, 39.3% for ABS/PP and 71.8% for ABS/PP/PP-g-MA. Even if the macroscopic simulated Von Mises stresses are similar for both microstructures, the local stress repartition leads to a more premature breakage in the case of ABS/PP/PP-g-MA blend. This observation can explain the result presented in Figure 5, the number of elements that have reached the critical breakage value is higher in the presence of PP-g-MA thus inducing progressively a reduction of stress in experiments for ABS/PP/PP-g-MA blend in comparison with ABS/PP.

**Figure 20:**
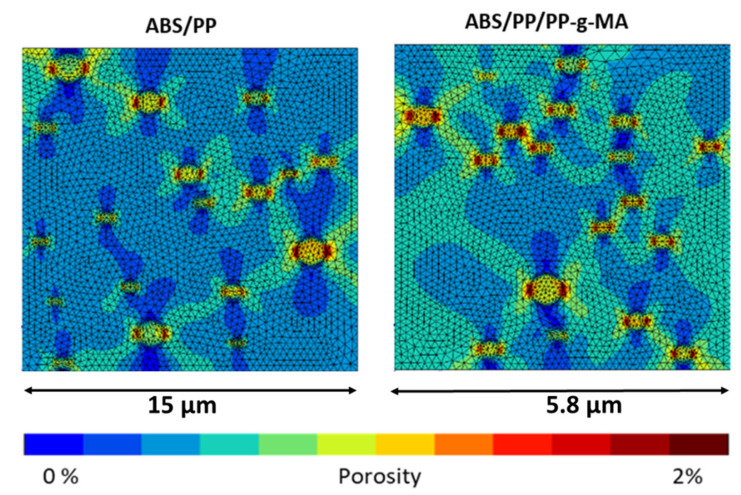
Porosity cartography at 1.7% of macroscopic strain in ABS/PP and ABS/PP/PP-g-MA composites.

**Figure 21:**
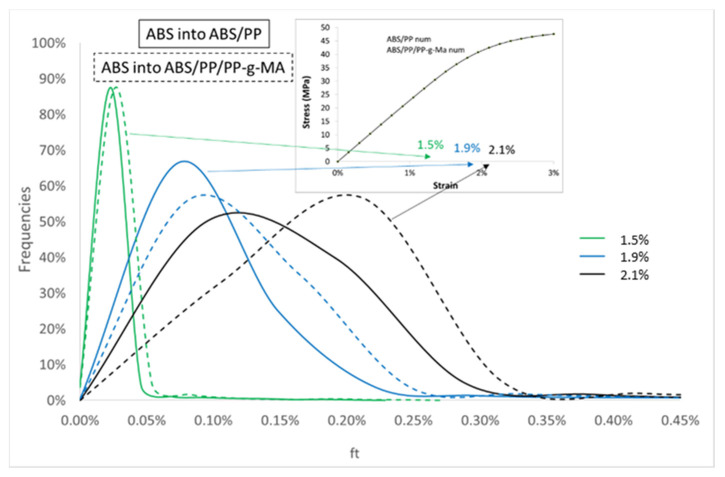
Frequencies of elements versus effective porosity fraction (see Equation (4)) for several macroscopic strains for ABS into ABS/PP and ABS/PP/PP-g-MA composites.

To complete this analysis cartography of porosity is given for 1.7% of macroscopic strain (Figure 20). In the PP nodules the porosity never exceeds 1% meaning that the PP never reaches the flow stress state. The porosity shows a concentration around the PP nodules in a direction perpendicular to the tensile direction. Even if these cartographies are quite similar, the distribution of porosity around the nodules seems different. The Figure 21 regroups the number of elements versus local porosities into ABS polymer for several macroscopic strains (1.5, 1.9, and 2.1%) for ABS/PP and ABS/PP/PP-g-MA blends. The more the macroscopic imposed strain increases, the more the shift between ABS/PP and ABS/PP/PP-g-MA is pronounced. The breakage is initiated at the interface ABS/PP by cavitation; this phenomenon is increased while reducing the size of nodules using a compatibilizing agent for example.

**Figure 22:**
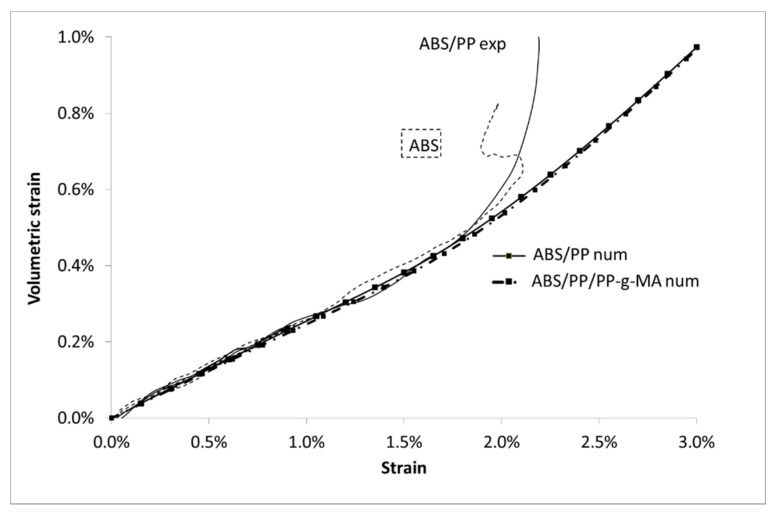
Volumetric strain vs. Lagrangian strain for ABS/PP composite, experiments, and modelling.

Regarding the volumetric strain, once again the prediction gives satisfactory results (Figure 22). Nevertheless, a deviation is observed from 1.7% of strain: the model underestimates the volume change. This result is in accordance with previous analysis, after 1.7% of macroscopic strain a large number of ABS elements have exceeded the ABS stress breakage. The comparison between experiments and numeric is therefore invalid. Experimentally, an interfacial decohesion between PP and ABS occurs thus increasing the volumetric strain explaining the observed deviation.

## 5. Conclusions

Polymer mechanical recycling is viewed nowadays as an efficient way to reduce plastic pollution and reduce the environmental footprint of its production from fossil resources. Sorting is primordial to achieve economic competitiveness since most polymers are incompatible. Residual impurities are however inevitable. Understanding the potential impact of these impurities is thus important to evaluate recyclability of end-of-life plastics. A first step is to work on a model blend. PP was incorporated at 4% in weight into ABS as they are two major constituents of WEEE. PP-g-MA was added to these blends with the aim to improve their mechanical performances. Experiments and predictive modelling were both used to assess first the ability of this blend to be reused at least in static applications, and second, the necessity of compatibilization.

The main conclusion of this paper is that static properties of ABS are almost not impaired by possible PP contamination at 4 wt% from impact tests with this impurity rate. Hence, this recycled material can be used as an ABS polymer, in the case of static application. Contrary to expectations, the addition of PP-g-MA, which was expected to improve ABS/PP adhesion and to reduce the size of the nodular phase, induced a negative effect on the ultimate stress. The main reason is that around the interfacial area, which increases as the nodules sizes decrease, the level of stress triaxiality in ABS is much higher. Locally, the ABS around PP nodules cavitates much more when nodules are even smaller. To go further in this predictive analysis, cohesive model zones should be used to mimic various interfacial compatibilities. An alternative could also be the use of an intermediate layer between phases with graduate properties [39]. The situation may be very different for dynamic applications as softer inclusions (PP are softer inclusions in ABS) can help to dissipate energy and accommodate stress.

## Figures and Tables

**Table 1:** Elastic properties of virgin polymers (ABS and PP) deduced from instrumented tensile tests at 1 and 10 mm/min.

Loading Speeds (mm/min)	1	1	10	10
Polymers	ABS	PP	ABS	PP
*E* (MPa)	2337 ± 9	1236 ± 25	2349 ± 12	1193 ± 42
*ν*	0.36 ± 0.01	0.39 ± 0.01	0.37 ± 0.01	0.43 ± 0.01

**Table 2:** Elastic and ultimate properties deduced from experiments for ABS, PP, ABS/PP composites at 10 mm/min.

	ABS	PP	ABS/PP	ABS/PP/PP-g-MA
*E* (MPa)	2349 ± 12	1193 ± 42	2329 ± 24	2212 ± 45
*ν*	0.37 ± 0.01	0.43 ± 0.005	0.37 ± 0.04	0.37 ± 0.02
$\sigma_{max}\left(\mathrm{MPa}\right)$	42 ± 5	31.7 ± 3	41 ± 4	39.4 ± 0.2
$\varepsilon_{u}\left(-\right)$	0.02 ± 0.01	0.18 ± 0.01	0.02 ± 0.01	0.027 ± 0.01

**Table 3:** Material model parameters for ABS and PP.

Material Properties	ABS	PP
*q*_1_(−), *q*_2_()	1, 1	1, 1
A()	0.53	0.23
*n*(), K (MPa.s)	2.5, 410	2.4, 51.2
$R_{0}\;\left(\mathrm{MPa}\right),\;Q_{1}\;\left(\mathrm{MPa}\right),\;b_{1}\left(-\right)$	27, 2, 210	11, 17, 58.3

**Table 4:** Percentage of elements in ABS with an equivalent stress higher than the ABS strength for ABS/PP and ABS/PP/PP-g-MA blends.

	% of Elements in ABS with an Equivalent Stress Higher than 43 Mpa	
	In ABS/PP	In ABS/PP/PP-g-MA
*ε*_macro_ = 0.3%	0.0	0.0
*ε*_macro_ = 1.5%	4.2	5.6
*ε*_macro_ = 1.9%	39.3	71.8
*ε*_macro_ = 2.1%	80.6	86.9
